# First records of genera *Chrysopera* Hampson, 1894 and *Entomogramma* Guenée, 1852 of the subfamily Erebinae (Lepidoptera, Erebidae) from South Korea

**DOI:** 10.3897/BDJ.12.e139471

**Published:** 2025-03-27

**Authors:** Hee Han, Sora Kim

**Affiliations:** 1 Jeonbuk National University, Jeonju, Republic of Korea Jeonbuk National University Jeonju Republic of Korea

**Keywords:** erebine moths, Korean peninsula, new record, owlet moths

## Abstract

**Background:**

The taxonomic status of two genera—*Chrysopera* and *Entomogramma*—within the subfamily Erebinae is still up for debate. This is because there are not many thorough phylogenetic studies based on large sampling and the Erebinae is one of the most speciose subfamilies of Lepidoptera with a high degree of diversity. They also lack in-depth comparative studies with morphologically related species.

**New information:**

Two little-known genera *Chrysopera* Hampson, 1894 and *Entomogramma* Guenée, 1852 are reported for the first time in Korea, based on *C.combinans* (Walker, 1858) and *E.fautrix* (Guenée, 1852). Illustrations of adults and genitalia are presented.

## Introduction

It is proposed that two genera, *Chrysopera* Hampson, 1894 and *Entomogramma* Guenée, 1852, should be classified within the subfamily Erebinae; however, their precise taxonomic position within this subfamily remains undetermined. This is due to the Erebinae’s high level of diversity, which is one of the most speciose subfamilies of Lepidoptera and the lack of comprehensive phylogenetic studies based on extensive sampling. The placement of these genera within the Erebinae is based on the accumulated morphological literature of several decades (Berio 1965, Holloway 2005, Homziak et al. 2016). *Entomogramma* has also been analysed in phylogenomic studies at the subfamily level (Homziak et al. 2019). However, there is a lack of specific taxonomic reviews or comparative studies with relative taxa of both genera. They are, therefore, considered to be under additional investigation.

The name *Chrysopera* was first used by Hampson (1894) with the species *Achaeacombinans* Walker as the type species. It was separated from *Achaea Hübner (1823)* into *Chrysopera* by Hampson (1894), however, without providing proper justification. After Hampson (1894), *Chrysopera* have not been taxonomically exchanged and *Avittapectinata*, later described by Holloway (1979), which had been compared to the genus *Pantura* (Holloway 2005), was considered more similar to the *Chrysopera*, based on features of male genitalia (Holloway 2005) and the *Chrysopera* became two species.

The *Entomogramma* was first established by Guenée (1852) with *Entomogrammafautrix* Guenée as a type specie with two other species: *E.torsa Guenée (1852)* and *E.pardus Guenée (1852)*. While proposing this genus, Guenée also divided it into two groups with the genus, the first group consisting of one species, *E.fautrix* and the second group consisting of *E.torsa* and *E.pardus*. He noted that the two groups are clearly distinct, based on the shape of the antennae and added that further taxonomic studies may be needed. Nine species have since been identified, mainly in Africa (Guenée 1852, Wallengren 1856, Walker 1858a, Walker 1865, Mabille 1880) and the Indo-Australian tropics (Guenée 1852, Felder et al. 1874, Pagenstecher 1890, Poole 1989, Haruta 1993, Hatura 1993). The *Entomogramma* was then placed in Hypopyrini by a phylogenomic study by Homziak et al. (2019), but further taxonomic work is required.

In this study, we report new records of two erebine genera, *Chrysopera* and *Entomogramma*, based on *C.combinans* and *E.fautrix* from Korea. We also provide taxonomic diagnoses for all species and distribution data, as well as illustrations of adults and genitalia. Furthermore, we present detailed descriptions of both species for the first time.

## Materials and methods

The materials utilised in this study, including slide vouchers, have been deposited at the following institute: Lab. Of Insect Phylogenetics & Evolution, Jeonbuk National University (IPE JBNU), Republic of Korea.

The procedure for genitalia preparation for vouchers was conducted in accordance with the methodology proposed by Kononenko and Han (2007). All dried specimens and slide vouchers of genitalia were examined under a Leica S9E microscope (Leica Microsystems, Germany) and images were taken with a Canon EOS 6D DSLR camera, Canon EF 100 mm F 2.8 L USM DSLR lens (Canon Inc., Japan) and a Leica LED5000 HDI (Leica Microsystems, Germany). Multi-stacked images were produced using Helicon Focus & Helicon Remote (HeliconSoft, Ukraine). The final images were processed using Adobe Photoshop Lightroom Classic and Adobe Photoshop 2024 (Adobe Systems, Inc., USA).

## Taxon treatments

### Chrysopera

Hampson, 1894

7412F823-2ACF-5CD2-80A1-4B681D0BF6AE

Chrysopera Hampson 1894: 493. Type Species. *Achaeacombinans* Walker, 1858

#### Diagnosis

This genus is similar to *Avitta Walker 1858b* in having brown ground colour of forewing, yellowish terminal part of hind-wing and dark brown abdomen. However, they can be easily distinguished by the following characteristics: male antennae narrowly bipectinated; hind-wing with broad yellow apex; eighth tergite of male abdomen broad, with strong sclerotisation of W-shape; eighth sternite of male trifid distally, with short central lobe. Male genitalia of this genus are also similar to those of *Asta Walker 1863*, *Pantura Moore 1885* and *Heoeugorna Hampson 1926*, but can be differentiated by the lack of scaphium and tegumen distinctly shorter than vinculum.

#### Distribution

Australasian, Oriental (Hampson 1894, Holloway 2005).

#### Notes

Diagnosis is based on Holloway (2005).

### Chrysopera combinans

(Walker, 1858)

8AB1EB86-D282-57E9-A6C7-5BB7A71FE095

Achaea combinans Walker 1858a: 1399. Type locality. Ceylon.
Achaea quadrilunata Pagenstecher 1890: 109. Type locality. E. Java.

#### Materials

- **Type status:** Other material. **Occurrence:** recordedBy: YB. Cha et al.; individualCount: 1; sex: female; lifeStage: adult; preparations: photograph; genitalia (slide no. HH0275 / Hee Han); occurrenceID: D37A1118-5631-56BA-8C22-C0BBD156FA4F; **Taxon:** kingdom: Animalia; phylum: Arthropoda; class: Insecta; order: Lepidoptera; family: Erebidae; genus: Chrysopera; specificEpithet: combinans; **Location:** country: South Korea; stateProvince: Jeollanam-do; locality: Wando-gun, Gunoe-myeon, Samdu-ri; decimalLatitude: 34.345528; decimalLongitude: 126.675500; **Event:** eventDate: 10 VII 2023

#### Description

**Figure 1.**
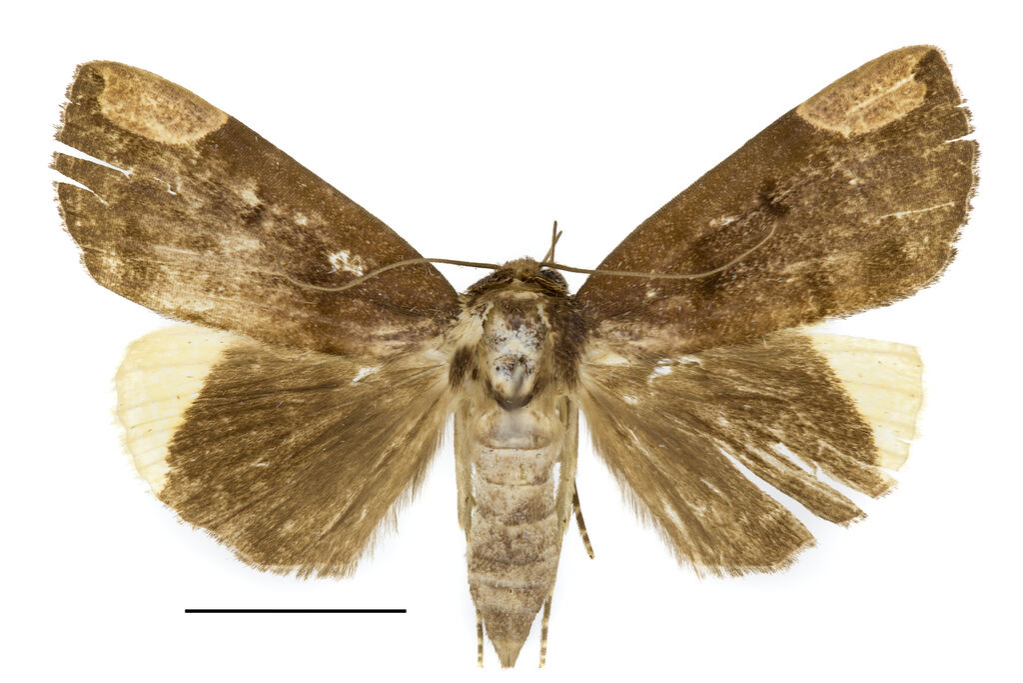
Adult of *Chrysoperacombinans*. Scale bar: 10 mm.

**Adult** (Fig. 1). Wing-span 39 mm. Antenna ciliated, reddish-brown. Head brown; frons yellowish-brown; labial palpus yellowish-brown; second segment dilatate, thick; third segment elongated. Thorax dark brown. Patagium and tegula dark brown. Femur of forelegs dark brown; tibia yellowish-brown; tarsus brown with bright scales on each segment. Femur of mid-legs brown; tibia furry, yellowish-brown, with a pair of spurs in the lower part; tarsus brown with bright scales on each segment, with spines. Hind-legs with femur yellowish-brown; tibia yellowish-brown, with two pairs of spurs in the middle part and lower part; tarsus yellowish-brown with bright scales on each segment, with spines. Ground colour of forewing brown; antemedial line indistinct, dark brown, arcuated; reniform blackish, bent; subapical spot light brown, distinct, semicircle, concave distally. Ground colour of hind-wing brown; apical area yellowish. Abdomen greyish-brown.

Male genitalia. Uncus slender, long, with pointed apex, deflexed; tegumen long, elongated, 1.5 times longer than uncus; valva broad, obovate; costa sclerotised, fused to valva; vinculum short, slightly broad, with round apex; aedeagus not shown (Holloway 2005).

**Figure 2.**
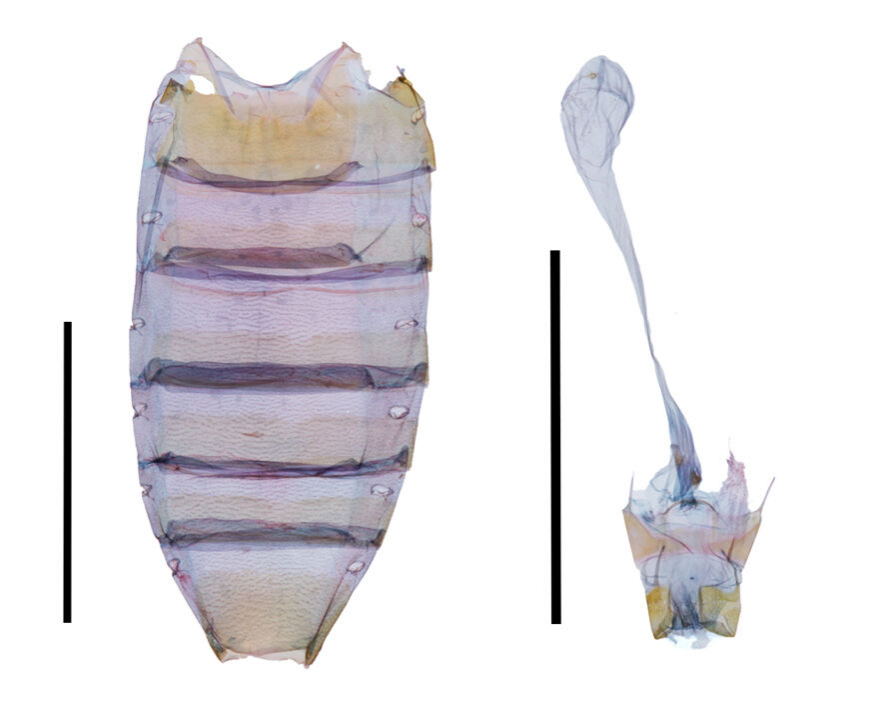
Female genitalia and abdomen of *Chrysoperacombinans*. Scale bar: 5 mm.

Female genitalia (Fig. 2). Papillae anales short, wide; apophyses posteriors 1.5 times longer than apophyses anteriores, both apophyses slender; ostium linear, slightly sclerotised; ductus bursae elongated with two sclerites near ostium; ductus seminalis short, elongated; corpus bursae elongated, with one small circular signum.

#### Diagnosis

This species is similar to *Avittabracteola* Holloway and *A.ochromarginata* Pagenstecher in having the following characters: brown forewing and dark brown hind-wing, with yellowish terminal part; dark brown abdomen (Holloway 2005). However, this species can be distinguished from *Avitta* species by the following characters: subapical spot light brown, distinct, semicircle, bent S-shape distally; apex of hind-wing yellowish.

#### Distribution

Palaearctic: Korea (new record); Oriental: India to Southeast Asia; Australasian: New Guinea, Queensland, the Solomons and Fiji (Holloway 2005).

#### Ecology

Hostplant: unknown.

#### Notes

Male genitalia description is based on fig. no. 330 of Holloway (2005). The report from Korea marks the highest latitude at which the species has ever been found. The specimen used in the present study has a 39 mm wing-span. However, earlier, Hampson (1894) measured 44 mm wing-span in this species.

### Entomogramma

Guenée, 1852

2B3ACC5B-DDDF-5C43-A29E-4F0FCE78ACA0

*Entomogramma Guenée 1852*: 203. Type Species. *Entomogrammafautrix* Guenée
Taramia Moore 1885: 153. Type Species. *Entomogrammatorsa* Guenée

#### Diagnosis

This genus is superficially similar to *Hypopyra Guenée 1852* in having entire brownish wings; forewing with blackish orbicular, subterminal line more distinct than all the others; pattern from forewing to hind-wing; underside of wings very distinct of two colours. However, they can be distinguished by the presence of the strong and fringed antennae of the *Entomogramma*.

#### Distribution

Afrotropic, Oriental (Guenée 1852).

### Entomogramma fautrix

Guenée, 1852

4D7C4ACB-DCAC-553C-88D6-69BDFF283205

Entomogramma fautrix Guenée 1852: 204. Type locality: Silhet.

#### Materials

- **Type status:** Other material. **Occurrence:** recordedBy: S. Kim et al.; individualCount: 1; sex: male; lifeStage: adult; preparations: photograph; genitalia (slide no. HH0278 / Hee Han); occurrenceID: B37E0731-A643-5629-824F-4216460FAA2F; **Taxon:** kingdom: Animalia; phylum: Arthropoda; class: Insecta; order: Lepidoptera; family: Erebidae; genus: Entomogramma; specificEpithet: fautrix; **Location:** country: South Korea; stateProvince: Jeollanam-do; locality: Wando-gun, Wando-eup, Jangjwa-ri; decimalLatitude: 34.352222; decimalLongitude: 126.701389; **Event:** eventDate: 27 VII 2022

#### Description

**Figure 3.**
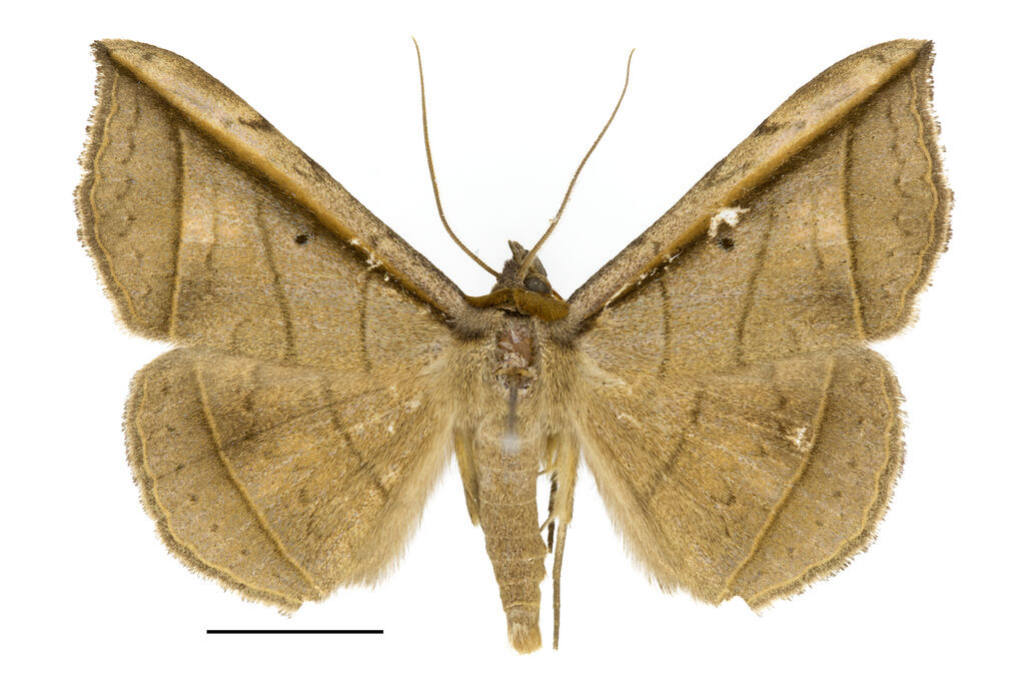
Adult of *Entomogrammafautrix*. Scale bar: 10 mm.

**Adult** (Fig. 3). Wing-span 48 mm. Antenna ciliated, reddish-brown, ivory alternately. Head blackish, tinged with brown scales; labial palpus blackish-brown, scattered yellow or brown scales; second segment plumpish, thicker than first and second segment; third segment small, elongated. Ground colour of thorax dark brown. Prothorax pale brownish, furry. Mesothorax yellowish-brown, with light grey. Metathorax brown. Patagium reddish-brown. Tegula yellowish-brown, with light grey fore part, with dark brownish dividing line. Femur and tibia of forelegs reddish-brown, furry; tarsus greyish-brown. Femur and tibia of mid-legs brown, furry, tibia with a pair of spurs in the lower part; tarsus greyish-brown, with spines. Femur of hind-legs yellowish-brown; tarsus brown, furry, with two pairs of spurs in the middle part and lower part; tibia yellowish-brown, with spines. Ground colour of forewing greenish brown; Distinct line straight, anteriorly reddish-brown, posteriorly dark brown, represented from apex to base along the subcostal vein. Six lines: basal one reddish-brown, straight; antemedial one reddish-brown, slightly leaning to apex; median one thin, wavy, sticking out to base at posterior part; postmedial one yellowish basally, weakly dark brown distally; subterminal one brown, diffused, zigzag; terminal one yellowish-brown, wavy. Cilia brown. Orbicular spot dark brown, small. Ground colour of hind-wing brown. Five lines: antemedial one straight; median one wavy; postmedial one straight; subterminal one brownish, dotted; terminal one yellowish. Cilia brown. Abdomen light brown.

**Figure 4.**
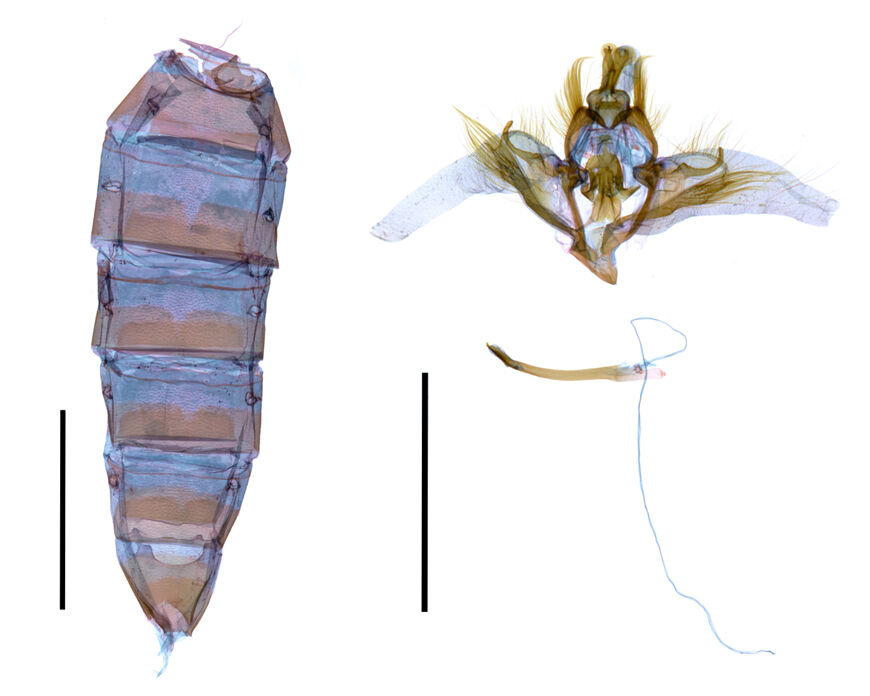
Male genitalia and abdomen of *Entomogrammafautrix*. Scale bar: 5 mm.

Male genitalia (Fig. 4). Uncus short, broad, bifurcated, with pointed apex, basal part broadened rectangular shape; tegumen curved, with long hairy setose; valva ear-shape, with strongly setose at sacculus, apex with one pointed spine; costa curved, slightly sclerotised, weakly setose; clasper weakly sclerotised; harpe sclerotised, curved, upturned; juxta plate-like, bifurcate in the middle part and lower part; hair pencil 2.5 times longer than valva, elastic; vinculum thin, with risen top and basal part; saccus inverted triangle shape; aedeagus slender, bent, 1.5 times longer than valva, with weakly sclerotised carina, apex pointed.

Female genitalia. Unknown.

#### Diagnosis

This species is similar to *E.torsa* in having forewing line from apex to base along the subcostal vein; line patterns from forewing to hind-wing (Guenée 1852). However, they can be distinguished by the following characters: the head with simple antennae; dark brown ground colour of wings; the forewing with distinct straight reddish line from apex to base along the subcostal vein and orbicular smaller than *E.torsa*.

#### Distribution

Palaearctic: Korea (new record); Oriental: Nepal, Bangladesh, India, Sri Lanka (Guenée 1852, Walker 1865, Haruta 1993).

#### Ecology

Hostplants *Pithecellobiumdulce* (Roxb.) Benth. [Leguminosae (M)] (Robinson et al. 2023).

#### Notes

This species is thought to be widely distributed from India to Southeast Asia, but is poorly documented (Holloway 2005). The discovery in South Korea represents the species’ northernmost known occurrence, applying that it is expanding northwards. The specimen used in the present study has a 48 mm wing-span. However, earlier, Guenée (1852) measured 55 mm wing-span in this species.

## Supplementary Material

XML Treatment for Chrysopera

XML Treatment for Chrysopera combinans

XML Treatment for Entomogramma

XML Treatment for Entomogramma fautrix

## References

[B12149602] Berio E. (1965). Le Catocaline Arficane a tibie spinose del Museo di Tervuren. Annali Del Museo Civico Di Storia Naturale Giacomo Doria.

[B12149611] Felder C., Felder R., Rogenhofer A. F. (1874). Reise der österreichischen Fregatte Novara um die Erde in den Jahren 1857, 1858, 1859 unter den Behilfen des Commodore B. von Wüllerstorf-Urbair. Zoologischer Theil. Band 2. Abtheilung 2. Lepidoptera. Rhopalocera. 4.

[B12149641] Guenée A. (1852). Histoire Naturelle des Insectes. Species Général des Lépidoptéres. Tome Septiéme. Noctuélites. Tome III..

[B12149649] Hampson G. F. (1894). The Fauna of British India, including Ceylon and Burma.

[B12210745] Hampson G. F. (1926). Descriptions of new Genera and Species of Lepidoptera Phalaenae of the subfamily Noctuinae (Noctuidae) in the British Museum (Natural History).

[B12149665] Haruta T. (1993). Moths of Nepal 2. Tinea.

[B12149674] Hatura T. (1993). Moths of Nepal 3. Tinea.

[B12149683] Holloway J. D. (1979). A survey of the Lepidoptera, biogeography and ecology of New Caledonia.

[B12149691] Holloway J. D. (2005). The moths of Borneo: Family Noctuidae, subfamily Catocalinae.

[B12149699] Homziak NICHOLAS T., Breinholt JESSE W., Kawahara AKITO Y. (2016). A historical review of the classification of Erebinae (Lepidoptera: Erebidae). Zootaxa.

[B12149708] Homziak Nicholas T., Breinholt Jesse W., Branham Marc A., Storer Caroline G., Kawahara Akito Y. (2019). Anchored hybrid enrichment phylogenomics resolves the backbone of erebine moths. Molecular Phylogenetics and Evolution.

[B12210711] Hübner J. (1823). Verzeichniss bekannter Schmettlinge.

[B12149718] Kononenko V. S., Han H. L., 2007 (2007). Atlas genitalia of Noctuidae in Korea (Lepidoptera).

[B12149747] Mabille P. (1880). Diagnoses Lepidopterum Malgassicorum. Annales de la Société entomologique de Belgique.

[B12149756] Moore F. (1885). The Lepidoptera of Ceylon.

[B12149764] Pagenstecher A. (1890). Beitrage zur Lepidopteren-Fauna des malayischen Archipels. Heterocera der Inseln Nias (bei Sumatra). Jahrbücher des Nassauischen Vereins für Naturkunde.

[B12149773] Poole R. W. (1989). Lepidopterorum catalogus (New Series) 118: Noctuidae.

[B12149781] Robinson G. S., Ackery P. R., Kitching I., Beccaloni G. W., Hernández. L. M. (2023). HOSTS – a Database of the World’s Lepidopteran Hostplants [Data set]. https://data.nhm.ac.uk/dataset/hosts.

[B12149790] Walker F. (1858). List of the specimens of lepidopterous insects in the collection of the British Museum, Part 14.

[B12210719] Walker F. (1858). List of the Specimens of Lepidopterous Insects in the Collection of the British Museum.

[B12210727] Walker F (1863). Catalogue of the Heterocerous Lepidopterous Insects collected at Sarawak, in Borneo, by Mr. A. R. Wallace, with Descriptions of New Species. Journal of the Proceedings of the Linnean Society of London..

[B12149798] Walker F. (1865). List of the specimens of lepidopterous Insects in the Collection of the British Museum, Part 33.

[B12149806] Wallengren H. D. J. (1856). Anteckningar I Zoologien. I. Kafferlandets Macrolepidopter-Fauna..

